# Personalized mechanical ventilation in acute respiratory distress syndrome

**DOI:** 10.1186/s13054-021-03686-3

**Published:** 2021-07-16

**Authors:** Paolo Pelosi, Lorenzo Ball, Carmen S. V. Barbas, Rinaldo Bellomo, Karen E. A. Burns, Sharon Einav, Luciano Gattinoni, John G. Laffey, John J. Marini, Sheila N. Myatra, Marcus J. Schultz, Jean Louis Teboul, Patricia R. M. Rocco

**Affiliations:** 1 Anesthesia and Intensive Care, San Martino Policlinico Hospital, IRCCS for Oncology and Neuroscience, Genoa, Italy; 2 Department of Surgical Sciences and Integrated Diagnostic (DISC), University of Genoa, Viale Benedetto XV 16, Genoa, Italy; 3 Pneumology and Intensive Care Medicine, University of São Paulo, São Paulo, Brazil; 4 Adult Intensive Care Unit, Albert Einstein Hospital, São Paulo, Brazil; 5 Department of Intensive Care, Austin Hospital, Melbourne, VIC Australia; 6 Department of Epidemiology and Preventive Medicine, Australian and New Zealand Intensive Care Research Centre, Monash University, Melbourne, VIC Australia; 7 Data Analytics Research and Evaluation Centre, The University of Melbourne and Austin Hospital, Melbourne, Australia; 8 Department of Intensive Care, Royal Melbourne Hospital, Melbourne, VIC Australia; 9 Department of Critical Care, The University of Melbourne, Melbourne, Australia; 10 Interdepartmental Division of Critical Care Medicine, University of Toronto, Toronto, ON Canada; 11 Unity Health Toronto-St. Michael’s Hospital, Li Ka Shing Knowledge Institute, Toronto, ON Canada; 12 Intensive Care Unit of the Shaare Zedek Medical Medical Centre, Hebrew University Faculty of Medicine, Jerusalem, Israel; 13 Department of Anaesthesiology, Emergency, and Intensive Care Medicine, University of Göttingen, Göttingen, Germany; 14 Anaesthesia and Intensive Care Medicine, University Hospital Galway, and School of Medicine, National University of Ireland, Galway, Ireland; 15 University of Minnesota and Regions Hospital, St. Paul, MN USA; 16 Department of Anaesthesiology, Critical Care and Pain, Tata Memorial Hospital, Homi Bhabha National Institute, Mumbai, India; 17 Mahidol Oxford Tropical Medicine Research Unit (MORU), Mahidol University, Bangkok, Thailand; 18 Service de Médecine Intensive-Réanimation, Hôpital Bicêtre, Inserm UMR S_999, AP-HP Université Paris-Saclay, Le Kremlin-Bicêtre, France; 19 Laboratory of Pulmonary Investigation, Carlos Chagas Filho Institute of Biophysics, Federal University of Rio de Janeiro, Rio de Janeiro, Brazil; 20 Department of Intensive Care, Amsterdam University Medical Centers, Amsterdam, The Netherlands; 21 Nuffield Department of Medicine, University of Oxford, Oxford, UK

**Keywords:** Tidal volume, Driving pressure, Transpulmonary pressure, Phenotype, Biomarkers, Chest computed tomography scan

## Abstract

A personalized mechanical ventilation approach for patients with adult respiratory distress syndrome (ARDS) based on lung physiology and morphology, ARDS etiology, lung imaging, and biological phenotypes may improve ventilation practice and outcome. However, additional research is warranted before personalized mechanical ventilation strategies can be applied at the bedside. Ventilatory parameters should be titrated based on close monitoring of targeted physiologic variables and individualized goals. Although low tidal volume (*V*_T_) is a standard of care, further individualization of *V*_T_ may necessitate the evaluation of lung volume reserve (e.g., inspiratory capacity). Low driving pressures provide a target for clinicians to adjust *V*_T_ and possibly to optimize positive end-expiratory pressure (PEEP), while maintaining plateau pressures below safety thresholds. Esophageal pressure monitoring allows estimation of transpulmonary pressure, but its use requires technical skill and correct physiologic interpretation for clinical application at the bedside. Mechanical power considers ventilatory parameters as a whole in the optimization of ventilation setting, but further studies are necessary to assess its clinical relevance. The identification of recruitability in patients with ARDS is essential to titrate and individualize PEEP. To define gas-exchange targets for individual patients, clinicians should consider issues related to oxygen transport and dead space. In this review, we discuss the rationale for personalized approaches to mechanical ventilation for patients with ARDS, the role of lung imaging, phenotype identification, physiologically based individualized approaches to ventilation, and a future research agenda.

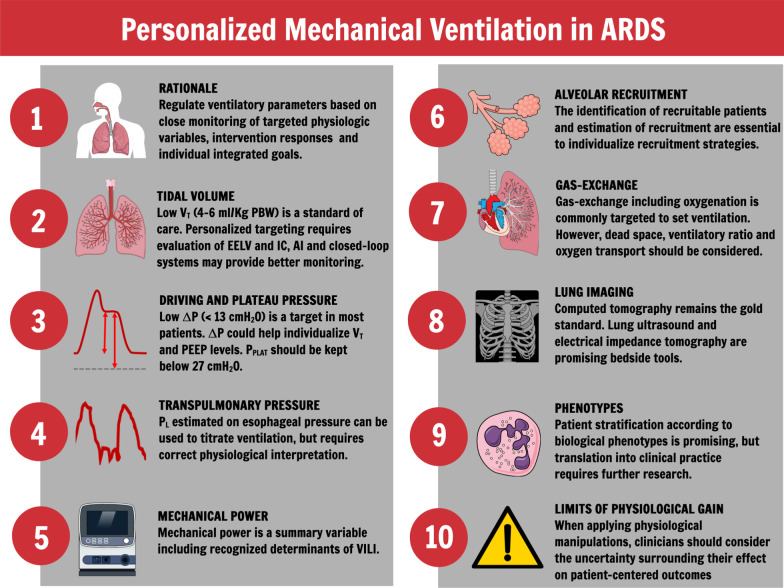

## Background

**Fig. 1:**
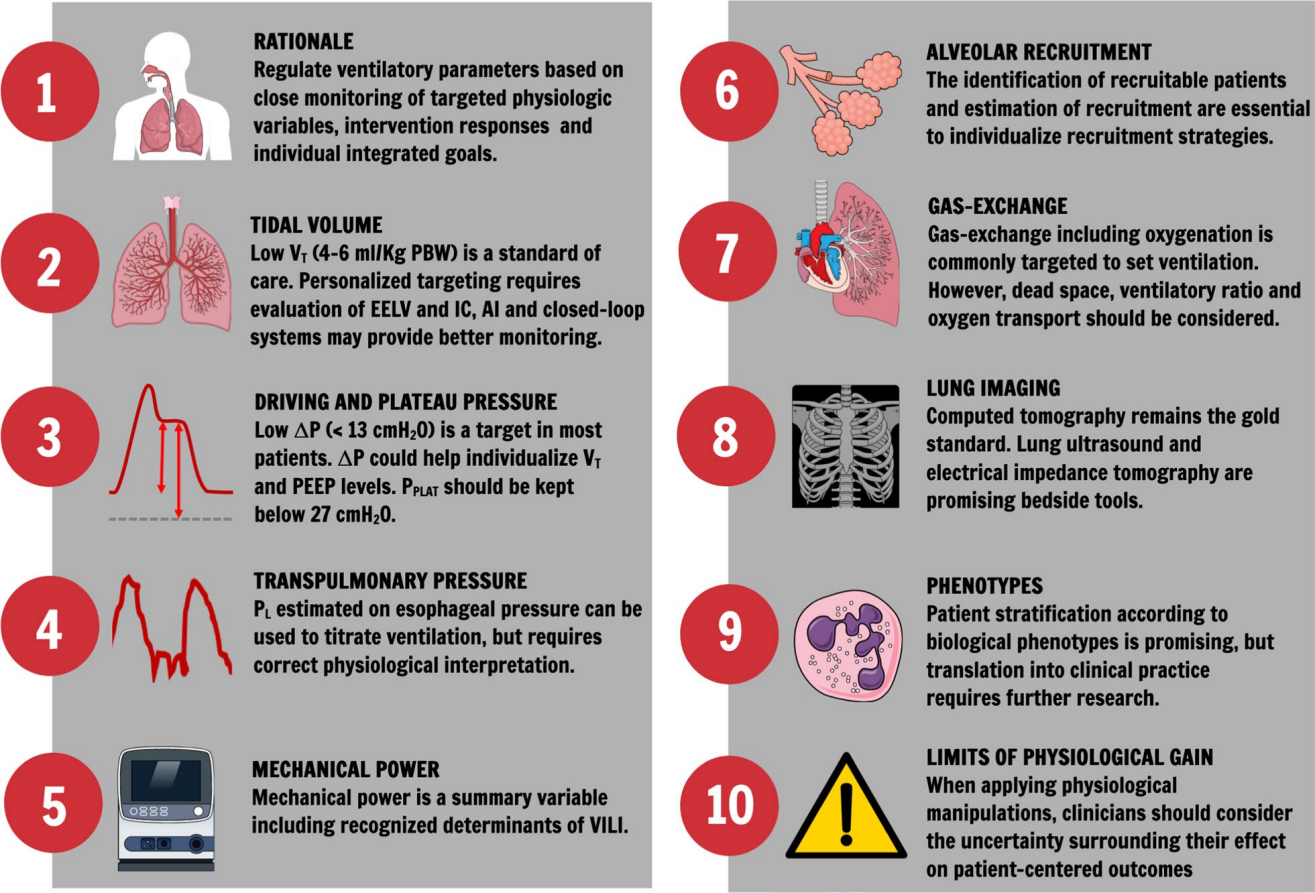
Summary of recommendations. *V*_T_: tidal volume; Δ*P*: driving pressure; PEEP: positive end-expiratory pressure; EELV: end-expiratory lung volume; IC: inspiratory capacity; AI: artificial intelligence; *P*_PLAT_: plateau pressure; VILI: ventilator-induced lung injury

Acute respiratory distress syndrome (ARDS) presents with a wide range of clinical and pathological characteristics [1, 2]. Mechanical ventilation is not a single disease-targeted therapy. Moreover, population-based data do not necessarily reflect individual patients with different phenotypes and co-morbidities [3, 4]. The development of treatments and strategies to manage patients with ARDS is complicated by its vast heterogeneity; thus, ARDS mortality remains high [5]. The present review discussed the rationale for personalized mechanical ventilation in ARDS, different ventilatory targets, the role of lung imaging, phenotype identification, physiologically based individualized approaches to ventilation, and a future research agenda. Figure 1 summarises the key points of this review.

### Rationale for personalized mechanical ventilation in ARDS

For clinicians, there is an understandable desire to standardize ventilatory management for patients with ARDS. Results from randomized clinical trials (RCTs) of interventions and strategies have been combined into meta-analyses to provide summary estimates of treatment effect to inform clinical practice and provide a starting point for a safe individualized approaches to mechanical ventilation [6]. Outcome studies that set thresholds for ventilatory variables based on mortality risk have not established a definitive causal link between the applied pattern, ventilator-induced lung injury (VILI), or death. At the bedside, clinicians seek to implement this evidence and adjust this “powerful instrument” by understanding physiologic mechanisms and possible consequences of ventilatory interventions [7]. Key goals are to relieve excessive workload of breathing and improve gas exchange, without impairing hemodynamics or incurring iatrogenic injury from intolerable pressures or inspired oxygen [8]. Notwithstanding, selective targeting of one of these objectives may collide with goals of another [9]. While using inflexible numerical ventilatory targets throughout all phases of ARDS is nonsensical, tailored ventilation based on careful functional monitoring and mechanistic understanding seems to be both more desirable and justified. Therefore, personalized mechanical ventilation based on lung physiology and response has laid the foundation to develop and inform “smarter” ventilation practices.

*Suggestion 1* Regulate the components of the ventilatory prescription based on close monitoring of targeted physiologic variables, intervention responses, and trends relevant to the integrated goals of treatment for the individual patient.

### Targeting tidal volume

Lung protective ventilation [targeting tidal volume (*V*_T_) of 4–6 mL/kg predicted body weight (PBW) to keep plateau pressure (*P*_PLAT_) below 30 cmH_2_O] is the current standard of care [10]. In healthy lungs, *V*_T_ can be titrated to PBW, since lung volumes are correlated with the PBW. By contrast in ARDS patients, lung volumes do not correlate closely with PBW due to heterogeneous distribution of lung disease. Thus, *V*_T_ should ideally be set according to end-expiratory lung volume (EELV) or inspiratory capacity (IC) measured at 30 cmH_2_O. At higher PEEP, EELV may change with respiratory system compliance (*C*_RS_), and setting of *V*_T_ according to EELV is not reliable. Higher PEEP may not affect or even reduce IC, thus limiting further increases in *V*_T_. At lower PEEP, IC correlates more closely with EELV, and *V*_T_ may be set by either IC or EELV [11]. Nevertheless, IC is easier for clinicians to measure at bedside compared to EELV [12]. The relationship between *V*_T_ and mortality is stronger in patients with lower *C*_RS_, suggesting the importance of targeting *V*_T_ in each patient according to the amount of aeration [3]. Processors within ventilators may automatically calculate the best *V*_T_, within safety ranges. For example, adaptive support ventilation, an automated closed-loop mode of ventilation, provides the best combination of *V*_T_ and respiratory rate (RR), to achieve the lowest work of breathing combined with the lowest driving pressure (Δ*P*) and may outperform healthcare professionals with respect to *V*_T_ titration [13]. Finally, the use of artificial intelligence to develop a personalized clinical decision support tool could provide needed support to bedside clinicians [14].

*Suggestion 2* Low *V*_T_ (4–6 ml/kg PBW) has become a standard of care. The personalized targeting of *V*_T_ may necessitate the evaluation of EELV or IC. Automated systems and artificial intelligence may enable better selection, monitoring, and control of optimal *V*_T._

### Targeting driving and plateau pressure

Driving pressure (Δ*P* = *V*_T_/*C*_RS_) estimates *V*_T_ adjusted to functional lung size and has been associated with mortality [15]. Recent studies suggest the importance of using Δ*P* to titrate *V*_T_ and/or PEEP in ARDS patients [16]. Assuming similar *C*_RS_, Δ*P* is directly correlated with *V*_T_. At low *C*_RS_, a reduced *V*_T_ is required to maintain Δ*P* within a safe range (< 13 cmH_2_O). Driving pressure may also be used to set PEEP since the best compromise between overinflation and recruitment is determined at the lowest Δ*P* [17]. A study analyzing two randomized ARDS trials found that a decrease in Δ*P* was associated with lower mortality compared to increased Pa_O2_/Fi_O2_ [18]. Several concerns exist regarding PEEP titration according to Δ*P* including (1) depending on the *V*_T_ used, the lowest Δ*P* may be achieved at different PEEP levels, (2) at higher *C*_RS_, compared to lower *C*_RS_, higher PEEP levels may achieve a lower Δ*P*, (3) the decrease in Δ*P* with PEEP may be associated with greater intratidal recruitment, (4) changes in chest wall compliance may affect the Δ*P* measurement, and (5) the presence of airway closure may confound the relationship between Δ*P* and PEEP. Although a causal effect has not been demonstrated, *P*_PLAT_ > 29 cmH_2_O and Δ*P* > 19 cmH_2_O in moderate/severe ARDS patients have been associated with increased hospital mortality, irrespective of the PEEP and *V*_T_ utilized [19]. Specific numerical values, however, may not apply to or be relevant for individual patients [20]. Additionally, the concept of transpulmonary Δ*P* during tidal ventilation has been gaining relevance recently and appears to hold potential for better guidance of protective mechanical ventilation.

*Suggestion 3* For most patients, Δ*P* should be targeted below 13 cmH_2_O. Although Δ*P* may help “individualize” *V*_T_ and PEEP settings, it is not clear whether Δ*P* is superior to other methods to set PEEP. Whenever possible, clinicians should aim to keep *P*_PLAT_ < 27 cmH_2_O.

### Targeting transpulmonary pressure

Transpulmonary pressure (PL), the distending force of the lung, is the difference between airway (*P*_AW_) and pleural pressure (*P*_PL_), with *P*_PL_ estimated by esophageal pressure (*P*_ES_) [21, 22]. During controlled mechanical ventilation, *P*_PL_ varies from non-dependent to dependent lung regions of the lung [23]. The absolute *P*_L_ gradient in the supine position primarily depends on lung weight as well as shape and mechanical properties of lung and chest wall. *P*_ES_ is a reasonable estimate of *P*_PL_ in the zone between the non-dependent and dependent lung regions. In ARDS, the superimposed pressure from non-dependent to dependent lung regions is 10 cmH_2_O, on average [24]; thus, *P*_PL_ is roughly *P*_ES_ + 5 cmH_2_O in dependent lung regions and *P*_ES_-5 cmH_2_O in non-dependent lung regions. When interpreting *P*_L_ from *P*_ES_ measurements, the absolute difference (not corrected) between *P*_AW_ and *P*_ES_ at end-inspiration or end-expiration represents the P_L_ in the middle lung, and the difference between end-inspiration and end-expiration in *P*_PL_ (Δ*P*_PL_) approximates Δ*P*_ES_. Elastance of respiratory system and chest wall may vary unpredictably and with changes in PEEP. In obese patients or those with increased intraabdominal pressure (*P*_PLAT_ above 27 cmH_2_O), a simplified formula may help estimate the required correction of *P*_PLAT_: *P*_PLAT_ target + (intraabdominal pressure-13 cmH_2_O)/2 [25, 26]. In mechanically ventilated non-obese patients, the average intraabdominal pressure is 13 cmH_2_O and half of intraabdominal pressure is transmitted to the thoracic cavity [27]. The following parameters have been suggested as potential targets for individualized mechanical ventilation when using *P*_L_ [28, 29]: (1) end-inspiratory *P*_L_ (non-dependent lung) below 15–20 cmH_2_O; (2) Δ*P*_L_ below 10–15 cmH_2_O; (3) PEEP set at end-expiratory *P*_L_ (dependent lung) equal to 0–6 cmH_2_O; and (4) P_L_ during recruitment maneuvers not to exceed 25 cmH_2_O [29, 30]. To date, RCTs evaluating the role of individualized PEEP set according to *P*_L_ at end-expiration and compared with low or high Pa_O2_/Fi_O2_ table have not shown beneficial effects on outcomes [31, 32].

*Suggestion 4* In patients with increased intraabdominal pressure or morbid obesity, *P*_L,_ may assist with individualizing ventilatory settings. The measurement of *P*_ES_ requires technical skills and physiologic interpretation to be applied by bedside clinicians.

### Targeting mechanical power

Mechanical power is defined as the amount of energy per unit of time and may vary within the span of an individual inflation or deflation half cycles by alteration in the flow profile [33]. The mechanical power computation is based on the following two equations of motion [34]:

1 $${\text{PRS}} = E_{{{\text{RS}}}} \cdot VT + \dot{V} \cdot R_{{{\text{aw}}}}$$

2 $${\text{PRS}} = E_{{{\text{RS}}}} \cdot VT + \dot{V} \cdot R_{{{\text{aw}}}} + {\text{PEEP}}$$

where *P*_*RS*_ is the change in respiratory system pressure, $\dot{V}$ is the inspiratory flow and *R*_aw_ the airway resistance. Equation 1 computes the *changes of pressure* from an undefined starting pressure, defining the *change* of the energy level stored in the respiratory system. Equation 2, defines the “absolute” energy level in the respiratory system with reference to its resting state. Multiplying the equations of motion by volume allows the computation of mechanical power for which several formulas are available [35–37].

A strong debate has ensued regarding PEEP and whether associated PEEP volume must be considered [38, 39] in the computation of mechanical power. Does adding a Δ*P* of 1 cm H_2_O to an already inflated lung (40 cmH_2_O of PEEP) produce a similar lung injury as when the same Δ*P* starts from 0 cm H_2_O PEEP? Supporters of the first equation of motion suggest that what really counts is the change. They claim that the energy required to climb one step on a staircase is the same regardless of which step one is starting from, as though the “rising the steps” occurs in a constant force field. In contrast, supporters of the second equation of motion claim that the force field varies with lung inflation. By analogy, it is as if a person climbed the stairs with an elastic band around the waist somehow anchored to the first step. Each step therefore requires more energy, and more energy results in more strain, and more strain results in more VILI. At present, there is a lack of agreement on the computation underpinning the calculation of mechanical power and determinants (PEEP levels, frequency, and lung size) that should be taken into consideration, and therefore, personalization is not possible. Although readily measured at the bedside, its value in VILI prediction remains controversial [40–42].

*Suggestion 5* Mechanical power is a summary construct that includes all of the important and well-recognized determinants of VILI. The same mechanical power value can be reached with different combinations of the above variables.

### Targeting alveolar recruitment

Ventilation strategies that target alveolar recruitment are based on the premise that a significant proportion of the volume loss within the ARDS “baby lung” [43] is due to alveolar edema and/or collapse, potentially be “recruited” to participate in gas exchange [44]. Recruitment maneuvers (RMs) typically apply higher airway pressures to open previously collapsed regions of lung. Higher PEEP levels are subsequently used to keep recruited alveoli open throughout the ventilation cycle. The safety of ventilation strategies targeting lung recruitment has been questioned. The ART trial [45] demonstrated that high-pressure stepwise lung recruitment maneuvers (to *P*_PLAT_ = 50–60 cmH_2_O) combined with higher PEEP titration increased patient mortality [45]. In contrast, the PHARLAP trial [46] tested a less aggressive recruitment strategy (*P*_PLAT_ ≤ 28 cmH_2_O), but was stopped early as the intervention group experienced higher rates of new cardiac dysrhythmias. A recent meta-analysis showed that at low *V*_T_, the routine use of higher PEEP and/or RMs did not reduce mortality among unselected ARDS patients [47]. In ARDS patients with a significant amount of collapsed lung, recruitment of these units could potentially reduce the pressure needed to accommodate a given *V*_T_ and the energy transmitted to individual lung units. Conversely, if these approaches do not recruit significantly collapsed alveoli, then the increased airway pressures could cause overdistension of open lung with negative cardiovascular effects. Monitoring the response to lung recruitment maneuvers at the bedside, regarding their effect on apparent lung compliance, is critical. A bedside approach to estimate recruitability has recently been proposed by abruptly releasing PEEP (from 15 to 5 cmH_2_O) with an increase in expired volume [48]. Briefly, the difference between expired volume and the volume predicted by compliance at low PEEP (or above airway opening pressure) estimates the recruited volume by PEEP. This recruited volume divided by the effective pressure change estimates the compliance of the recruited lung; the ratio of the compliance of the recruited lung to the compliance at low PEEP measures the recruitment-to-inflation ratio. The recruitment-to-inflation ratio may help to identify ARDS patients who are recruitable at the bedside.

*Suggestion 6* The identification of recruitability in ARDS patients, such as by the estimation of alveolar recruitment at bedside, is essential to personalize the use of recruitment strategies. However, systematic use of RMs is not associated with better outcome.

### Targeting gas exchange

The use the PaO_2_/FiO_2_-PEEP table has become a standard by which clinicians set PEEP. The largest trial investigating the effects of different ventilatory strategies in ARDS used a PaO_2_/FiO_2_-PEEP table to set PEEP. However, PaO_2_/FiO_2_ depends on FiO_2_ and PEEP levels affect cardiac output (CO). Recently, the concept of “keeping the lung at rest with permissive atelectasis and minimal oxygenation targets” has been proposed [49]. Oxygen consumption (VO_2_) is dependent on oxygen delivery (DO_2_); consequently, maximizing DO_2_ might be considered an alternative therapeutic goal for managing ARDS patients [50, 51]. Both a meta-analysis [52] and RCTs [53, 54] have failed to show benefits on outcomes from maximizing DO_2_. In ARDS patients, it is important to individually maintain an adequate DO_2_ and hence adequate CO as opposed to targeting supra-normal values of DO_2_. Central venous oxygen saturation (ScvO_2_), as a reflection of the VO_2_/DO_2_ balance, may be a good marker of CO adequacy [55]. However, ARDS associated with sepsis or marked systemic inflammation impairs oxygen extraction capabilities and renders ScvO_2_ uninterpretable [56]. In these conditions, the difference between central venous and arterial CO_2_ pressure (PCO_2_ gap) is useful since it is not affected by altered oxygen extraction. A PCO_2_ gap higher than 6 mmHg is indicative of inadequate DO_2_ and is increased in the presence of shock. Physiologic dead space is the portion of each *V*_T_ that does not take part in gas exchange and represents a good “global index” of the efficiency of the lung function being strongly associated with outcome and helpful for PEEP setting [57, 58]. However, dead space is not routinely measured in critical care practice, because of the difficulties in interpreting capnograms and the different calculation methods. The ventilation ratio and P_ET_CO_2_/PaCO_2_ ratio, though less precise, appear to be an excellent surrogate for physiologic dead space [59, 60].

*Suggestion 7* Gas exchange, including oxygenation, is a widely used parameter to set mechanical ventilation in ARDS. Physiologic dead space and the ventilation ratio should be also considered, taking into account *the balance* between aeration and perfusion. The measurement of PCO_2_ gap is important to individually identify inadequate DO_2._

### Targeting lung imaging

Different imaging techniques have been proposed to personalize mechanical ventilation strategies. Lung ultrasound (LUS) and electric impedance tomography (EIT) may be performed at bedside, while chest computed tomography (CT) scan require transportation of patients outside of the intensive care unit (ICU). Chest CT scan allows the detection of relevant parenchymal alterations including the amount and regional lung distribution of hyperinflated, aerated, and non-aerated lung tissue [61]. Two main chest CT phenotypes have been proposed [62]: (1) lobar attenuations (e.g., focal findings) associated with minimal loss of lung volume, less increase in lung weight, a linear pressure–volume curve of the respiratory system, with minimal alveolar recruitment and increased hyperinflation in response to increased PEEP, and (2) diffuse or patchy attenuations (e.g., non-focal findings) associated with major loss of lung volume, marked increases in lung weight, a curvilinear pressure–volume curve of the respiratory system, with greater alveolar recruitment and less hyperinflation in response to an increase in PEEP. Recruitment maneuvers yield less hyperinflation in patients with non-focal compared to focal chest CT morphology [63]. Chest CT morphology, in turn, may be associated with different biomarkers [64] and impaired alveolar clearance [5]. A recent clinical trial [65] found no differences in outcome between standard lung–protective ventilation and personalized ventilation based on the morphology of consolidations, the “open–lung strategy” consisting of high PEEP with RMs and rescue prone positioning in patients with non-focal ARDS while low PEEP without RMs and early prone positioning in focal ARDS. Patients that were misclassified in the personalized ventilation group had higher mortality compared to patients that received the intended ventilation strategy.

Dual-energy CT scan has been recently used to investigate ventilation perfusion relationships at different ventilation settings in COVID-19 patients [66]. LUS has been proposed as an alternative tool to monitor lung morphology at bedside [67], for several reasons: (1) LUS and chest CT findings are well correlated with regard to aeration [68] and identifying patients with focal or non-focal ARDS morphology [69], (2) LUS may influence clinical decision making related to the individualized mechanical ventilation management [70, 71], and (3) worsening aeration in several LUS regions, associated with deterioration of respiratory mechanics and blood gases, requires prompt reevaluation of mechanical ventilation settings. Notwithstanding, sensitivity of LUS to detect alveolar recruitment is variable across studies [68]. Conversely, EIT assesses regional differences between inspiratory and expiratory aeration and permits in-depth characterization of ARDS phenotypes at the bedside [72]. PEEP, when set according to EIT, may aid in optimizing lung recruitment and homogeneity of ventilation [73]. Technological development by EIT may also quantitatively estimate regional lung perfusion based on first-pass kinetics of a bolus of hypertonic saline contrast [74, 75]. To evaluate lung morphology and the potential for recruitment, ARDS patients should ideally undergo chest or lung imaging at different pressures.

*Suggestion 8* Imaging techniques may help to better identify different lung morphology and response to ventilation strategies. Chest CT allows detailed and quantitative analysis of overdistended lung: normally aerated, poorly aerated and non-aerated tissue, as well as consolidated and atelectatic components. LUS and EIT are promising tools for clinicians to use at the bedside.

### Targeting biological phenotypes

ARDS is characterized by different pathogenetic pathways leading to similar clinical presentations postulated to represent distinct phenotypes, which may enable precision therapy. To date, two different ARDS phenotypes (hyperinflammatory and hypoinflammatory) have been identified that differ in response to therapy and outcomes. Post hoc latent class analysis of a panel of blood biomarkers for inflammation, endothelial injury, and coagulopathy combined with clinical variables has revealed two phenotypes in 5 RCTs [76–80]. Similarly, post hoc cluster analysis of a set of biomarkers for inflammation, endothelial injury and coagulopathy without clinical variables also revealed two phenotypes in an observational study [81]. Of interest, the phenotypes had a differential or even opposite response to PEEP, fluid management, and simvastatin treatment [76–78, 80]. Recently, point-of-care breath testing introduced the possibility for targeted exhaled breath analysis to be used as a bedside test and potentially a diagnostic tool for timely ARDS detection [82]. Genome-wide association studies sequencing of hundreds-of-thousands to millions of DNA variants (single nucleotide polymorphisms) may help to identify individual patients who display a phenotype or trait that may be more or less amenable to a specific treatments [83]. The personalized approach to treatment based on identification of phenotypes according to different biomarkers is based on two main assumptions: (1) the patient’s phenotype can be correctly identified and (2) treatment needs to be individually targeted and effective for specific phenotypes. However, the personalized approach to treatment based on phenotypes may be associated with better outcomes in diseases with a single identifiable factor or etiology, such as cancer. Conversely, in ARDS, the disease etiology and progression may be linked to multiple factors. Thus, the efficacy of a specific treatment is not assured even if individual phenotypes are identified correctly. Care must be taken to refrain from prematurely positive interpretations of secondary analyses of previously collected data, which have not been validated in prospective cohorts or RCTs. To date, no mechanical ventilation guidelines have included statements regarding different ARDS phenotypes.

*Suggestion 9* The stratification of ARDS patients according to different phenotypes is promising but awaits clinical confirmation before it can be translated into to the clinical setting.

### The seduction of short-term physiological gains

In daily practice, critical care physicians use physiological data to aid them in guiding and adjusting therapies. However, clinicians are not able to accurately predict the medium- or long-term consequences of physiologically based therapy on patient-centered outcomes. Clinicians often fall prey to celebrating immediate physiological gains under the pretense that they will translate into improvements in desired outcomes. In this regard, physiological gains can be seductive. When these gains are achieved, clinicians feel validated (immediacy bias) regardless of the downstream future effects which are unknown. When anticipated gains are not achieved, clinicians engage in attribution bias-blaming severe and unresponsive illness. Short-term physiological gains are an elusive concept. For example, in an ARDS patient on PEEP = 8 cmH_2_O and FiO_2_ = 0.6 to maintain a PaO_2_ = 60 mmHg, increasing PEEP to 14 cmH_2_O may achieve the same PaO_2_ at an FiO_2_ = 0.5. Some clinicians may see that as physiological success. However, apportioning “value” to this increase in PEEP prioritizes lung mechanics and lung physiology because they can be measured. However, the impact of the increased PEEP on renal function, gastrointestinal permeability, or the brain typically remains unmeasured and unknown. This is a fundamental problem with physiology: it measures whether a specific intervention affects a specific set of physiological parameters. However, it does not assess “other” unmeasured or unintended physiological consequences. Thus, physiologically based personalized therapy, although clearly important at the extremes of illness, become problematic at intermediate levels of illness where safety has typically been established. Historically, the pursuit of perceived physiological success in the belief that it would lead to subsequent clinical success has often proved to be disappointing with notable examples including intensive insulin therapy to normalize glycemia [84]; drotrecogin alpha to normalize activated protein C levels [85]; colloid resuscitation to increase intravascular volume [86]; decompressive craniectomy to lower intracranial pressure in diffuse cerebral injury [87]; hypothermia for out of hospital cardiac arrest [88], early parenteral or enteral nutrition to achieve early full caloric intake [89]; glutamine therapy to correct glutamine deficiency [90]; fluid bolus resuscitation in septic African children [91]. All this does not imply that physiology should not be used to guide therapies in patients with ARDS. However, clinicians should be aware that improvement in physiological variables during individualized targeted therapy does not necessarily imply clinical safety or improved outcomes.

*Suggestion 10* When applying physiological manipulations, clinicians should consider the uncertainty surrounding their subsequent effect on patient-centered outcomes.

### Future research agenda

Personalized mechanical ventilation in ARDS merits further research on specific targets: (1) characterize biomarkers profiles and responses to specific treatments associated with pulmonary or extrapulmonary insults, pulmonary inflammation, and lung physiology; (2) the use of mechanical power and *P*_L_; and (3) identification of patient’s phenotype according to the biomarkers of epithelial and endothelial cell damage, inflammation, and extracellular matrix. The investment of personalized mechanical ventilation is high and will require investment of both personnel and resources, including experimental and clinical trials.

## Conclusions

A personalized mechanical ventilation approach based on lung physiology and morphology, ARDS etiology, lung imaging as well as identification of biological phenotypes may improve and individualize future mechanical ventilation practice. Additional research is warranted before personalized mechanical ventilation strategies can be applied at the bedside of ARDS patients.

## Data Availability

Not applicable.

## Abbreviations

ARDS: Acute respiratory distress syndrome
RCT: Randomized clinical trial
VILI: Ventilator induced lung injury
*V*_T_: Tidal volume
PBW: Predicted body weight
*P*_PLAT_: Plateau pressure heterogeneous distribution of lung disease
EELV: End-expiratory lung volume
IC: Inspiratory capacity
PEEP: Positive end-expiratory pressure
*C*_RS_: Respiratory system compliance
RR: Respiratory rate
Δ*P*: Driving pressure
PL: Transpulmonary pressure
*P*_AW_: Airway pressure
*P*_PL_: Pleural pressure
*P*_ES_: Esophageal pressure regions
Δ*P*_L_: Transpulmonary driving pressure
*P*_RS_: Respiratory system pressure
$$\dot{V}$$: Inspiratory flow
*R*_aw_: Airway resistance
RM: Recruitment maneuver
FiO_2_: Inspired oxygen fraction
CO: Cardiac output
VO_2_: Oxygen consumption
DO_2_: Oxygen delivery
ScvO_2_: Central venous oxygen saturation
PCO_2_ gap: Central venous and arterial CO_2_ pressure difference
P_ET_CO_2_: End-tidal CO_2_
LUS: Lung ultrasound
EIT: Electric impedance tomography
CT: Computed Tomography
ICU: Intensive care unit

## References

[1] Ashbaugh DG, Bigelow DB, Petty TL, Levine BE (1967). Acute respiratory distress in adults. Lancet Lond Engl.

[2] Thille AW, Peñuelas O, Lorente JA, Fernández-Segoviano P, Rodriguez J-M, Aramburu J-A (2017). Predictors of diffuse alveolar damage in patients with acute respiratory distress syndrome: a retrospective analysis of clinical autopsies. Crit Care Lond Engl.

[3] Goligher EC, Costa ELV, Yarnell CJ, Brochard LJ, Stewart TE, Tomlinson G (2021). Effect of lowering Vt on mortality in acute respiratory distress syndrome varies with respiratory system elastance. Am J Respir Crit Care Med.

[4] Bos LDJ, Artigas A, Constantin J-M, Hagens LA, Heijnen N, Laffey JG (2021). Precision medicine in acute respiratory distress syndrome: workshop report and recommendations for future research. Eur Respir Rev Off J Eur Respir Soc..

[5] Bellani G, Laffey JG, Pham T, Fan E, Brochard L, Esteban A (2016). Epidemiology, patterns of care, and mortality for patients with acute respiratory distress syndrome in intensive care units in 50 countries. JAMA.

[6] Djulbegovic B, Guyatt GH (2017). Progress in evidence-based medicine: a quarter century on. Lancet.

[7] Brochard L, Hedenstierna G (2016). Ten physiologic advances that improved treatment for ARDS. Intensive Care Med.

[8] Battaglini D, Sottano M, Ball L, Robba C, Rocco PRM, Pelosi P. Ten golden rules for individualized mechanical ventilation in acute respiratory distress syndrome. J Intensive Med. 2021. 10.1016/j.jointm.2021.01.003 PMC7919509 36943812

[9] Silva PL, Gama de Abreu M (2018). Regional distribution of transpulmonary pressure. Ann Transl Med..

[10] Putensen C, Theuerkauf N, Zinserling J, Wrigge H, Pelosi P (2009). Meta-analysis: ventilation strategies and outcomes of the acute respiratory distress syndrome and acute lung injury. Ann Intern Med.

[11] Hubmayr RD (2011). Point: is low tidal volume mechanical ventilation preferred for all patients on ventilation?. Yes Chest.

[12] Mattingley JS, Holets SR, Oeckler RA, Stroetz RW, Buck CF, Hubmayr RD (2011). Sizing the lung of mechanically ventilated patients. Crit Care Lond Engl.

[13] Botta M, Wenstedt EFE, Tsonas AM, Buiteman-Kruizinga LA, van Meenen DMP, Korsten HHM (2021). Effectiveness, safety and efficacy of INTELLiVENT–adaptive support ventilation, a closed–loop ventilation mode for use in ICU patients—a systematic review. Expert Rev Respir Med.

[14] Mamandipoor B, Frutos-Vivar F, Peñuelas O, Rezar R, Raymondos K, Muriel A (2021). Machine learning predicts mortality based on analysis of ventilation parameters of critically ill patients: multi-centre validation. BMC Med Inform Decis Mak.

[15] Amato MBP, Meade MO, Slutsky AS, Brochard L, Costa ELV, Schoenfeld DA (2015). Driving pressure and survival in the acute respiratory distress syndrome. N Engl J Med.

[16] Barbas CSV, Palazzo RF (2018). Should we titrate mechanical ventilation based on driving pressure?—yes. Ann Transl Med.

[17] Chen L, Jonkman A, Pereira SM, Lu C, Brochard L (2021). Driving pressure monitoring during acute respiratory failure in 2020. Curr Opin Crit Care.

[18] Sakr Y, François B, Solé-Violan J, Kotfis K, Jaschinski U, Estella A (2021). Temporal changes in the epidemiology, management, and outcome from acute respiratory distress syndrome in European intensive care units: a comparison of two large cohorts. Crit Care Lond Engl.

[19] Villar J, Martín-Rodríguez C, Domínguez-Berrot AM, Fernández L, Ferrando C, Soler JA (2017). A Quantile analysis of plateau and driving pressures: effects on mortality in patients with acute respiratory distress syndrome receiving lung-protective ventilation. Crit Care Med.

[20] Costa ELV, Slutsky A, Brochard LJ, Brower R, Serpa-Neto A, Cavalcanti AB (2021). Ventilatory variables and mechanical power in patients with acute respiratory distress syndrome. Am J Respir Crit Care Med.

[21] Yoshida T, Brochard L (2018). Esophageal pressure monitoring: why, when and how?. Curr Opin Crit Care.

[22] Akoumianaki E, Maggiore SM, Valenza F, Bellani G, Jubran A, Loring SH (2014). The application of esophageal pressure measurement in patients with respiratory failure. Am J Respir Crit Care Med.

[23] Pelosi P, Goldner M, McKibben A, Adams A, Eccher G, Caironi P (2001). Recruitment and derecruitment during acute respiratory failure: an experimental study. Am J Respir Crit Care Med.

[24] Pelosi P, D’Andrea L, Vitale G, Pesenti A, Gattinoni L (1994). Vertical gradient of regional lung inflation in adult respiratory distress syndrome. Am J Respir Crit Care Med.

[25] Yoshida T, Amato MBP, Grieco DL, Chen L, Lima CAS, Roldan R (2018). Esophageal manometry and regional transpulmonary pressure in lung injury. Am J Respir Crit Care Med.

[26] Chiumello D, Carlesso E, Cadringher P, Caironi P, Valenza F, Polli F (2008). Lung stress and strain during mechanical ventilation for acute respiratory distress syndrome. Am J Respir Crit Care Med.

[27] Regli A, Pelosi P, Malbrain MLNG (2019). Ventilation in patients with intra-abdominal hypertension: what every critical care physician needs to know. Ann Intensive Care.

[28] Tilmont A, Coiffard B, Yoshida T, Daviet F, Baumstarck K, Brioude G (2021). Oesophageal pressure as a surrogate of pleural pressure in mechanically ventilated patients. ERJ Open Res..

[29] Mauri T, Yoshida T, Bellani G, Goligher EC, Carteaux G, Rittayamai N (2016). Esophageal and transpulmonary pressure in the clinical setting: meaning, usefulness and perspectives. Intensive Care Med.

[30] Fan E, Del Sorbo L, Goligher EC, Hodgson CL, Munshi L, Walkey AJ (2017). An Official American Thoracic Society/European Society of Intensive Care Medicine/Society of Critical Care Medicine Clinical Practice Guideline: mechanical ventilation in adult patients with acute respiratory distress syndrome. Am J Respir Crit Care Med.

[31] Talmor D, Sarge T, Malhotra A, O’Donnell CR, Ritz R, Lisbon A (2008). Mechanical ventilation guided by esophageal pressure in acute lung injury. N Engl J Med.

[32] Beitler JR, Sarge T, Banner-Goodspeed VM, Gong MN, Cook D, Novack V (2019). Effect of titrating positive end-expiratory pressure (PEEP) With an esophageal pressure-guided strategy vs an empirical high PEEP-Fio2 strategy on death and days free from mechanical ventilation among patients with acute respiratory distress syndrome: a randomized clinical trial. JAMA.

[33] Marini JJ, Rocco PRM, Gattinoni L (2020). Static and dynamic contributors to ventilator-induced lung injury in clinical practice. Pressure, energy, and power. Am J Respir Crit Care Med.

[34] Gattinoni L, Tonetti T, Cressoni M, Cadringher P, Herrmann P, Moerer O (2016). Ventilator-related causes of lung injury: the mechanical power. Intensive Care Med.

[35] Becher T, van der Staay M, Schädler D, Frerichs I, Weiler N (2019). Calculation of mechanical power for pressure-controlled ventilation. Intensive Care Med.

[36] Giosa L, Busana M, Pasticci I, Bonifazi M, Macrì MM, Romitti F (2019). Mechanical power at a glance: a simple surrogate for volume-controlled ventilation. Intensive Care Med Exp.

[37] Silva PL, Ball L, Rocco PRM, Pelosi P (2019). Power to mechanical power to minimize ventilator-induced lung injury?. Intensive Care Med Exp.

[38] Huhle R, Serpa Neto A, Schultz MJ, Gama de Abreu M (2018). Is mechanical power the final word on ventilator-induced lung injury?—no. Ann Transl Med..

[39] Vasques F, Duscio E, Pasticci I, Romitti F, Vassalli F, Quintel M (2018). Is the mechanical power the final word on ventilator-induced lung injury?—we are not sure. Ann Transl Med.

[40] Marini JJ, Gattinoni L, Rocco PR (2020). Estimating the damaging power of high-stress ventilation. Respir Care.

[41] Rocco PRM, Silva PL, Samary CS, Hayat Syed MK, Marini JJ (2020). Elastic power but not driving power is the key promoter of ventilator-induced lung injury in experimental acute respiratory distress syndrome. Crit Care Lond Engl.

[42] Marini JJ, Rocco PRM (2020). Which component of mechanical power is most important in causing VILI?. Crit Care Lond Engl.

[43] Gattinoni L, Marini JJ, Pesenti A, Quintel M, Mancebo J, Brochard L (2016). The, “baby lung” became an adult. Intensive Care Med.

[44] Caironi P, Carlesso E, Cressoni M, Chiumello D, Moerer O, Chiurazzi C (2015). Lung recruitability is better estimated according to the Berlin definition of acute respiratory distress syndrome at standard 5 cm H2O rather than higher positive end-expiratory pressure: a retrospective cohort study. Crit Care Med.

[45] Writing Group for the Alveolar Recruitment for Acute Respiratory Distress Syndrome Trial (ART) Investigators, Cavalcanti AB, Suzumura ÉA, Laranjeira LN, Paisani DM, Damiani LP, et al. Effect of lung recruitment and titrated positive end-expiratory pressure (PEEP) vs low PEEP on mortality in patients with acute respiratory distress syndrome: a randomized clinical trial. JAMA 2017;318:1335–45. 10.1001/jama.2017.14171 PMC5710484 28973363

[46] Hodgson CL, Cooper DJ, Arabi Y, King V, Bersten A, Bihari S (2019). Maximal recruitment open lung ventilation in acute respiratory distress syndrome (PHARLAP): a phase II, multicenter, randomized, controlled trial. Am J Respir Crit Care Med.

[47] Ball L, Serpa Neto A, Trifiletti V, Mandelli M, Firpo I, Robba C (2020). Effects of higher PEEP and recruitment manoeuvres on mortality in patients with ARDS: a systematic review, meta-analysis, meta-regression and trial sequential analysis of randomized controlled trials. Intensive Care Med Exp.

[48] Chen L, Del Sorbo L, Grieco DL, Junhasavasdikul D, Rittayamai N, Soliman I (2020). Potential for lung recruitment estimated by the recruitment-to-inflation ratio in acute respiratory distress syndrome. A clinical trial. Am J Respir Crit Care Med.

[49] Pelosi P, Rocco PRM, Gama de Abreu M (2018). Close down the lungs and keep them resting to minimize ventilator-induced lung injury. Crit Care Lond Engl..

[50] Danek SJ, Lynch JP, Weg JG, Dantzker DR (1980). The dependence of oxygen uptake on oxygen delivery in the adult respiratory distress syndrome. Am Rev Respir Dis.

[51] Krachman SL, Lodato RF, Morice R, Gutierrez G, Dantzker DR (1994). Effects of dobutamine on oxygen transport and consumption in the adult respiratory distress syndrome. Intensive Care Med.

[52] Steltzer H, Hiesmayr M, Mayer N, Krafft P, Hammerle AF (1994). The relationship between oxygen delivery and uptake in the critically ill: is there a critical or optimal therapeutic value? A meta-analysis. Anaesthesia.

[53] Hayes MA, Timmins AC, Yau EH, Palazzo M, Hinds CJ, Watson D (1994). Elevation of systemic oxygen delivery in the treatment of critically ill patients. N Engl J Med.

[54] Gattinoni L, Brazzi L, Pelosi P, Latini R, Tognoni G, Pesenti A (1995). A trial of goal-oriented hemodynamic therapy in critically ill patients. SvO2 collaborative group. N Engl J Med.

[55] Teboul J-L, Saugel B, Cecconi M, De Backer D, Hofer CK, Monnet X (2016). Less invasive hemodynamic monitoring in critically ill patients. Intensive Care Med.

[56] Vieillard-Baron A, Matthay M, Teboul JL, Bein T, Schultz M, Magder S (2016). Experts’ opinion on management of hemodynamics in ARDS patients: focus on the effects of mechanical ventilation. Intensive Care Med.

[57] Morales-Quinteros L, Schultz MJ, Bringué J, Calfee CS, Camprubí M, Cremer OL (2019). Estimated dead space fraction and the ventilatory ratio are associated with mortality in early ARDS. Ann Intensive Care.

[58] Fengmei G, Jin C, Songqiao L, Congshan Y, Yi Y (2012). Dead space fraction changes during PEEP titration following lung recruitment in patients with ARDS. Respir Care.

[59] Bonifazi M, Romitti F, Busana M, Palumbo MM, Steinberg I, Gattarello S (2021). End-tidal to arterial PCO2 ratio: a bedside meter of the overall gas exchanger performance. Intensive Care Med Exp.

[60] Ferluga M, Lucangelo U, Blanch L (2018). Dead space in acute respiratory distress syndrome. Ann Transl Med.

[61] Pesenti A, Musch G, Lichtenstein D, Mojoli F, Amato MBP, Cinnella G (2016). Imaging in acute respiratory distress syndrome. Intensive Care Med.

[62] Puybasset L, Gusman P, Muller JC, Cluzel P, Coriat P, Rouby JJ (2000). Regional distribution of gas and tissue in acute respiratory distress syndrome. III. Consequences for the effects of positive end-expiratory pressure. CT Scan ARDS Study Group. Adult respiratory distress syndrome. Intensive Care Med.

[63] Constantin J-M, Grasso S, Chanques G, Aufort S, Futier E, Sebbane M (2010). Lung morphology predicts response to recruitment maneuver in patients with acute respiratory distress syndrome. Crit Care Med.

[64] Mrozek S, Jabaudon M, Jaber S, Paugam-Burtz C, Lefrant J-Y, Rouby J-J (2016). Elevated plasma levels of sRAGE are associated with nonfocal CT-based lung imaging in patients with ARDS: a prospective multicenter study. Chest.

[65] Constantin J-M, Jabaudon M, Lefrant J-Y, Jaber S, Quenot J-P, Langeron O (2019). Personalised mechanical ventilation tailored to lung morphology versus low positive end-expiratory pressure for patients with acute respiratory distress syndrome in France (the LIVE study): a multicentre, single-blind, randomised controlled trial. Lancet Respir Med.

[66] Ball L, Robba C, Herrmann J, Gerard SE, Xin Y, Mandelli M (2021). Lung distribution of gas and blood volume in critically ill COVID-19 patients: a quantitative dual-energy computed tomography study. Crit Care.

[67] Temsah M-H, Al-Sohime F, Alhaboob A, Al-Eyadhy A, Aljamaan F, Hasan G (2021). Adverse events experienced with intrahospital transfer of critically ill patients: a national survey. Medicine.

[68] Chiumello D, Mongodi S, Algieri I, Vergani GL, Orlando A, Via G (2018). Assessment of lung aeration and recruitment by CT scan and ultrasound in acute respiratory distress syndrome patients. Crit Care Med.

[69] Costamagna A, Pivetta E, Goffi A, Steinberg I, Arina P, Mazzeo AT (2021). Clinical performance of lung ultrasound in predicting ARDS morphology. Ann Intensive Care.

[70] Xirouchaki N, Kondili E, Prinianakis G, Malliotakis P, Georgopoulos D (2014). Impact of lung ultrasound on clinical decision making in critically ill patients. Intensive Care Med.

[71] Smit MR, Pisani L, de Bock EJE, van der Heijden F, Paulus F, Beenen LFM (2021). Ultrasound versus computed tomography assessment of focal lung aeration in invasively ventilated ICU patients. Ultrasound Med Biol.

[72] Scaramuzzo G, Spinelli E, Spadaro S, Santini A, Tortolani D, Dalla Corte F (2020). Gravitational distribution of regional opening and closing pressures, hysteresis and atelectrauma in ARDS evaluated by electrical impedance tomography. Crit Care Lond Engl.

[73] Scaramuzzo G, Spadaro S, Dalla Corte F, Waldmann AD, Böhm SH, Ragazzi R (2020). Personalized positive end-expiratory pressure in acute respiratory distress syndrome: comparison between optimal distribution of regional ventilation and positive transpulmonary pressure. Crit Care Med.

[74] Borges JB, Suarez-Sipmann F, Bohm SH, Tusman G, Melo A, Maripuu E (2012). Regional lung perfusion estimated by electrical impedance tomography in a piglet model of lung collapse. J Appl Physiol.

[75] Hentze B, Muders T, Luepschen H, Maripuu E, Hedenstierna G, Putensen C (2018). Regional lung ventilation and perfusion by electrical impedance tomography compared to single-photon emission computed tomography. Physiol Meas.

[76] Calfee CS, Delucchi K, Parsons PE, Thompson BT, Ware LB, Matthay MA (2014). Subphenotypes in acute respiratory distress syndrome: latent class analysis of data from two randomised controlled trials. Lancet Respir Med.

[77] Famous KR, Delucchi K, Ware LB, Kangelaris KN, Liu KD, Thompson BT (2017). Acute respiratory distress syndrome subphenotypes respond differently to randomized fluid management strategy. Am J Respir Crit Care Med.

[78] Sinha P, Delucchi KL, Thompson BT, McAuley DF, Matthay MA, Calfee CS (2018). Latent class analysis of ARDS subphenotypes: a secondary analysis of the statins for acutely injured lungs from sepsis (SAILS) study. Intensive Care Med.

[79] Delucchi K, Famous KR, Ware LB, Parsons PE, Thompson BT, Calfee CS (2018). Stability of ARDS subphenotypes over time in two randomised controlled trials. Thorax.

[80] Calfee CS, Delucchi KL, Sinha P, Matthay MA, Hackett J, Shankar-Hari M (2018). Acute respiratory distress syndrome subphenotypes and differential response to simvastatin: secondary analysis of a randomised controlled trial. Lancet Respir Med.

[81] Bos LD, Schouten LR, van Vught LA, Wiewel MA, Ong DSY, Cremer O (2017). Identification and validation of distinct biological phenotypes in patients with acute respiratory distress syndrome by cluster analysis. Thorax.

[82] Hagens LA, Verschueren ARM, Lammers A, Heijnen NFL, Smit MR, Nijsen TME (2021). Development and validation of a point-of-care breath test for octane detection. Analyst.

[83] Du M, Garcia JGN, Christie JD, Xin J, Cai G, Meyer NJ (2021). Integrative omics provide biological and clinical insights into acute respiratory distress syndrome. Intensive Care Med.

[84] NICE-SUGAR Study Investigators, Finfer S, Chittock DR, Su SY-S, Blair D, Foster D, et al. Intensive versus conventional glucose control in critically ill patients. N Engl J Med. 2009;360:1283–97. 10.1056/NEJMoa0810625 19318384

[85] Ranieri VM, Thompson BT, Barie PS, Dhainaut J-F, Douglas IS, Finfer S (2012). Drotrecogin alfa (activated) in adults with septic shock. N Engl J Med.

[86] Perner A, Haase N, Guttormsen AB, Tenhunen J, Klemenzson G, Åneman A (2012). Hydroxyethyl starch 130/0.42 versus Ringer’s acetate in severe sepsis. N Engl J Med.

[87] Cooper DJ, Rosenfeld JV, Murray L, Arabi YM, Davies AR, D’Urso P (2011). Decompressive craniectomy in diffuse traumatic brain injury. N Engl J Med.

[88] Dankiewicz J, Cronberg T, Lilja G, Jakobsen JC, Levin H, Ullén S (2021). Hypothermia versus normothermia after out-of-hospital cardiac arrest. N Engl J Med.

[89] Casaer MP, Mesotten D, Hermans G, Wouters PJ, Schetz M, Meyfroidt G (2011). Early versus late parenteral nutrition in critically ill adults. N Engl J Med.

[90] Heyland D, Muscedere J, Wischmeyer PE, Cook D, Jones G, Albert M (2013). A randomized trial of glutamine and antioxidants in critically ill patients. N Engl J Med.

[91] Maitland K, Kiguli S, Opoka RO, Engoru C, Olupot-Olupot P, Akech SO (2011). Mortality after fluid bolus in African children with severe infection. N Engl J Med.

